# TGF-beta1诱导人肺腺癌PC9细胞上皮-间质转化的研究

**DOI:** 10.3779/j.issn.1009-3419.2010.01.06

**Published:** 2010-01-20

**Authors:** 慧君 张, 雷 张, 和勇 王, 晓峰 陈

**Affiliations:** 200433 上海，上海市肺科医院 Department of Pneumosurgery, Shanghai Pulmonary Hospital, Shanghai, 200433, China

**Keywords:** TGF-β1, 肺腺癌PC9细胞, EMT, TGF-β1, Lung adencarcinoma PC9 cells, EMT

## Abstract

**背景与目的:**

研究表明上皮-间质转化（epithelial-mesenchymal transition, EMT）不仅参与胚胎形成与发育，而且参与肿瘤侵袭转移。此外，人转化生长因子-β1（transforming growth factor-beta1, TGF-β1）已被证实为肿瘤EMT的主要诱导剂。本研究旨在探讨TGF-β1诱导人肺腺癌PC9细胞发生EMT及其对PI3K/AKT信号通道的影响。

**方法:**

将体外培养的PC9细胞用不同浓度TGF-β1处理48 h，相差倒置显微镜下观察细胞形态学变化；Western blot和细胞免疫荧光验证EMT相关标记蛋白表达变化。同时，采用Western blot方法检测AKT和P-AKT的表达水平。

**结果:**

TGF-β1可诱导PC9细胞向间质型细胞形态转化，并上调间质标记蛋白Fibronectin的表达及下调P-AKT的表达。

**结论:**

TGF-β1可诱导PC9细胞发生EMT，并影响PI3K/AKT信号通道。

## Abstract (en)

**Background and objective:**

It has been proven that epithelial-mesenchymal transition (EMT) not only correlated with embryonic development but also could promote tumor invasion and metastasis. Transforming growth factor beta-1 (TGF-β1) has been identified as the main inducer of tumor EMT. The aim of this study was to investigate the effects of TGF-β1 on EMT and PI3K/AKT signaling pathway in lung adencarcinoma PC9 cells.

**Methods:**

Cultured PC9 cells were treated with different concentrations of TGF-β1 for 48 h. The morphological changes were observed under phase-contrast microscopy; EMT relative marker protein changes were assessed by Western blot and immunoflurescence staining. In addition, the expression of AKT and P-AKT were also measured by Western blot.

**Results:**

The data showed that TGF-β1 could induce PC9 morphological alteration from epithelial to mesenchymal and upregulate the expression of mesenchymal maker protein Fibronectin. Obviously, the expression of P-AKT was downregulated by TGF-β1 treatment for 48 h.

**Conclusion:**

TGF-β1 might induce EMT of PC9 cells, accompanied by the changes of PI3K/AKT signaling pathway.

在恶性肿瘤中，肺癌仍是目前最常见的死亡原因。许多肺癌患者在就诊时已发生转移，据统计90%的肺癌患者死亡由转移引起^[1]^，最近研究^[2, 3]^表明上皮-间质转化（epithelial-mesenchymal transition, EMT）在肿瘤侵袭转移过程中起重要作用。

EMT是指上皮细胞失去其上皮特征，获得间质特性的过程，它不仅参与胚胎形态的形成、心脏的发育和慢性退行性纤维化，而且促进肿瘤侵袭转移^[4]^。此外，细胞外信号的生长因子如HGF、EGF、TGF-β、IGF、VEGF等可经过不同的信号通道诱导细胞发生EMT，已证实PI3K/AKT信号通路参与EMT发生^[5]^。本文旨在研究TGF-β1诱导人肺腺癌PC9细胞发生EMT及对PI3K/AKT信号通路的影响。

## 1 材料与方法

### 1.1 材料

人肺腺癌PC9细胞为上海肺科医院肺癌免疫研究室常规传代培养；TGF-β1购自R&D公司；单克隆兔抗人E-cadherin、多克隆兔抗人AKT和单克隆小鼠抗人p-AKT均购自Cell Signaling Technology公司；单克隆小鼠抗人Fibronectin和Vimentin购自Santa Cruz公司；辣根酶标记羊抗小鼠或抗兔IgG购自Abgent公司；F1TC标志的羊抗兔IgG购自Upstates公司；DMEM购自Gibco公司。

### 1.2 细胞培养

PC9细胞在37 ℃、5%CO_2_、饱和湿度条件下用含10%新生牛血清、100 U/mL青霉素、100 μg/mL庆大霉素、低糖-DMEM培养基中培养。用0.25%胰酶消化传代。细胞培养至融合70%-80%后，用无血清培养基饥饿过夜，再加入TGF-β1处理48 h，按实验要求分组。

### 1.3 细胞形态学观察

将PC9细胞传代于6孔板并用无血清培养基饥饿过夜，经不同浓度（0 ng/mL、1 ng/mL、5 ng/mL）TGF-β1处理48 h后在相差显微镜下观察细胞形态变化并拍照。

### 1.4 Western blot检测

不同浓度（0 ng/mL、1 ng/mL、5 ng/mL）TGF-β1处理细胞48 h后，培养瓶中的贴壁细胞用4 ℃的PBS洗涤后转移到EP管，12 000 rpm离心5 min，弃去上清，将收集到的细胞加入细胞裂解液裂解后，取上清液-20 ℃储存。上样，80 V电泳积聚蛋白，100 V电压使蛋白分离并转膜，5%脱脂牛奶封闭1 h。用单克隆兔抗人的E-cadherin（1:1 000）、单克隆小鼠抗人的Fibronectin和Vimentin（1:100）、多克隆兔抗人AKT和单克隆小鼠抗人p-AKT（1:500）分别与膜接触4 ℃孵育过夜后，用TBST在脱色摇床下洗膜5 min×3次，在加辣根酶标记羊抗小鼠或抗兔IgG，室温下孵育1 h后，用TBST洗膜5 min×3次，加入ECL化学发光显示剂在室温下反应1 min后，用X光片曝光，然后定影、显影。

### 1.5 细胞免疫荧光检测

以每孔1×10^4^个细胞接种于6孔培养板中预置的载玻片上，细胞贴壁爬片，用无血清培养基饥饿过夜，加入不同浓度（0 ng/mL、1 ng/mL、5 ng/mL）TGF-β1处理48 h后，取出载玻片，用0.4%多聚甲醛固定15 min，加入单克隆兔抗人的E-cadherin（1:200）、单克隆小鼠抗人Fibronectin和Vimentin（1:50）4 ℃过夜，加FITC标志的羊抗兔IgG和Cy3标志的羊抗小鼠IgG（1:50），37 ℃下孵育1 h，缓冲甘油封固，荧光显微镜下观察并拍片。

## 2 结果

### 2.1 TGF-β1诱导PC9细胞形态学变化观察

PC9细胞在不同浓度（0 ng/mL、1 ng/mL、5 ng/mL）TGF-β1处理48 h后，通过相差显微镜观察，未处理的PC9细胞呈不典型上皮细胞形态，经TGF-β1刺激后，大部分细胞形态明显拉长，呈现明显的间质细胞形态，且5 ng/mL TGF-β1组比1 ng/mL组更为明显。此外，细胞间连接也变得更疏松（图 1）。

**1:**
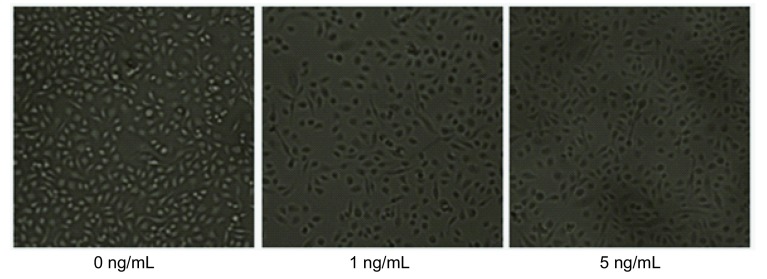
PC9细胞经不同浓度（0 ng/mL、1 ng/mL、5 ng/mL）TGF-β1处理48 h后的形态学变化 Morphologic changes of PC9 cells induced by different concentrations (0 ng/mL, 1 ng/mL, 5 ng/mL) of TGF-β1 for 48 h

### 2.2 TGF-β1对PC9细胞上皮及间质标记蛋白表达的影响不同浓度

（0 ng/mL、1 ng/mL、5 ng/mL）TGF-β1处理PC9细胞48 h后，Western blot显示TGF-β1并不影响PC9细胞上皮标记蛋白E-cadherin和间质标记蛋白Vimentin的表达。然而，间质标记蛋白Fibronectin的表达量却随TGF-β1浓度的增加也相应增加（图 2）。另外通过细胞免疫荧光也证实了Western blot的结果（图 3）。

**2:**
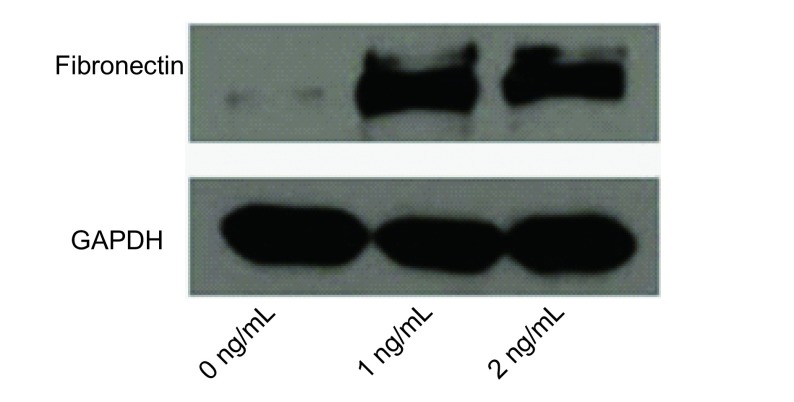
Western blot检测PC9细胞EMT相关标记蛋白Fibronectin表达变化 The expression change of EMT related marker protein Fibronectin in PC9 cells was assessed by Western blot

**3:**
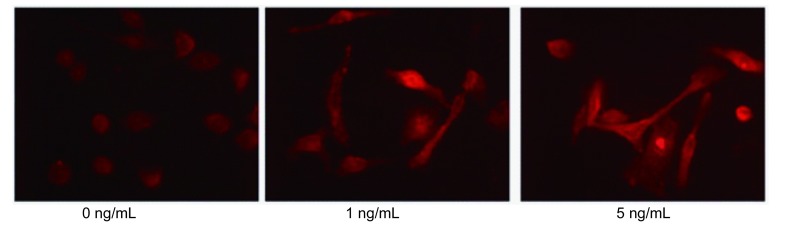
细胞免疫荧光检测PC9细胞EMT相关标记蛋白Fibronectin表达变化 The expression change of EMT related marker protein Fibronectin in PC9 cells was detected by immunoflurescence staining

### 2.3 TGF-β1对PI3K/AKT信号通路的影响

PC9细胞经不同浓度（0 ng/mL、1 ng/mL、5 ng/mL）TGF-β1处理48 h后，Western blot显示P-AKT经TGF-β1处理后其表达量降低，且1 ng/mL组和5 ng/mL组P-AKT表达量减少大致相仿（图 4）。

**4:**
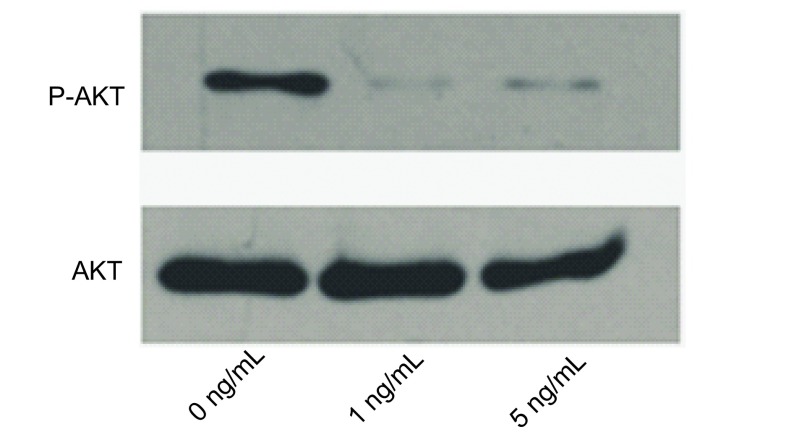
Western blot检测TGF-β1对PC9细胞AKT和P-AKT的表达 The expression of AKT and P-AKT in PC9 cells was assessed by Western blot

## 3 讨论

肿瘤转移是恶性肿瘤进展的重要步骤，也是恶性肿瘤患者死亡的重要原因。因此研究肿瘤转移的分子机制对抑制恶性肿瘤转移、提高肿瘤患者生存率至关重要。肿瘤转移是一个复杂的、多步骤的过程，首先肿瘤细胞脱离原发灶，通过血管生成和基底膜重建进入循环系统，但进入循环系统的肿瘤细胞必须克服循环系统的各种障碍才能到达转移部位。肿瘤转移涉及多种调控机制，其中促进肿瘤细胞转移的重要机制之一就是EMT^[6, 7]^。

Greenburg等^[8]^指出培养中的上皮细胞可获得间质细胞特性，最早提出了EMT发生的证据。在胚胎形成、肿瘤发生发展及某些纤维化疾病的过程中，TGF-β作为EMT的主要诱导剂备受关注^[9]^。TGF-β1作为TGF-β家族的重要成员之一，在肿瘤发生发展及某些纤维化疾病过程中已被证实为EMT的主要诱导剂^[10-13]^。但是，对人肺腺癌PC9细胞来说，TGF-β1能否诱导PC9细胞发生EMT及对PI3K/AKT信号通路影响又如何？目前尚无相关报道。

TGF-β1诱导PC9细胞发生EMT首先在细胞形态方面被证实^[7]^。Kasai等^[11]^指出肺腺癌A549细胞在TGF-β1作用下，由典型卵石状的上皮表型转化成纤维细胞样的间质表型，同时相邻细胞间连接也变得疏松^[11]^。Yan等^[14]^用低氧处理肝癌细胞发生EMT，细胞诱导形态结果与Kasai相似。本研究结果显示未处理的PC9细胞呈不典型的上皮细胞形态，经TGF-β1处理后大部分细胞形态较未处理组明显拉长，相邻细胞间连接也变得疏松。为了进一步证实经TGF-β1处理的PC9细胞可发生EMT，我们首先用Western blot验证EMT相关标记蛋白，结果显示TGF-β1处理PC9细胞既没有下调上皮标记蛋白E-cadherin的表达，也没上调间质标记蛋白Vimentin的表达，仅仅是上调了间质标记蛋白Fibronectin的表达，细胞免疫荧光也证实了间质标记蛋白Fibronectin表达上调。Shintani等^[15]^指出培养中的肿瘤细胞仅发生不完全EMT，从严格意义上讲我们并不能观察到真正的EMT^[15]^。Voulgari等^[16]^指出发生不完全EMT的肿瘤细胞，其分子标记物和细胞特征可发生不同程度的改变^[16]^。因此，在本研究中，TGF-β1可诱导肺腺癌PC9细胞发生EMT，且为不完全EMT。

TGF-β可通过酪氨酸激酶受体迅速激活PI3K/AKT信号通路^[17, 18]^。PI3K激酶调节亚基已被证实与TβRI和TβRII受体相关，并可提高TGF-β的激活效果^[19]^。PI3K/AKT信号通路的阻断可抑制TGF-β诱导的细胞形态学变化，并使a-SAM表达下调和E-cadherin表达上调^[20, 21]^。由此可见，在EMT过程中，TGF-β在PI3K/AKT信号通路的激活中起重要作用。Chao等^[22]^指出经TGF-β处理的肿瘤细胞的p-AKT表达量在4 h时达到高峰，之后其表达量逐渐减低。我们用TGF-β1处理PC9细胞48 h后p-AKT的表达量较未处理组明显减少，而1 ng/mL组和5 ng/mL组的减少量并无差别，可推测PC9细胞在TGF-β1的刺激下，p-AKT的表达随时间的变化而发生改变，但与TGF-β1浓度关系不大。

总而言之，TGF-β1可诱导肺腺癌PC9细胞发生不完全EMT，并影响PI3K/AKT信号通路。已证实PI3K/AKT信号通路参与胃癌的侵袭转移^[23]^，那么此通路对肺腺癌PC9细胞的侵袭转移能力又有何影响，还需进一步研究。
